# Neural Correlates of Motor Recovery after Robot-Assisted Training in Chronic Stroke: A Multimodal Neuroimaging Study

**DOI:** 10.1155/2021/8866613

**Published:** 2021-06-09

**Authors:** Cheng Chen, Kai Yuan, Xin Wang, Ahsan Khan, Winnie Chiu-wing Chu, Raymond Kai-yu Tong

**Affiliations:** ^1^Department of Biomedical Engineering, The Chinese University of Hong Kong, Shatin, Hong Kong; ^2^Department of Imaging and Interventional Radiology, The Chinese University of Hong Kong, Shatin, Hong Kong

## Abstract

Stroke is a leading cause of motor disability worldwide, and robot-assisted therapies have been increasingly applied to facilitate the recovery process. However, the underlying mechanism and induced neuroplasticity change remain partially understood, and few studies have investigated this from a multimodality neuroimaging perspective. The current study adopted BCI-guided robot hand therapy as the training intervention and combined multiple neuroimaging modalities to comprehensively understand the potential association between motor function alteration and various neural correlates. We adopted EEG-informed fMRI technique to understand the functional regions sensitive to training intervention. Additionally, correlation analysis among training effects, nonlinear property change quantified by fractal dimension (FD), and integrity of M1-M1 (M1: primary motor cortex) anatomical connection were performed. EEG-informed fMRI analysis indicated that for iM1 (iM1: ipsilesional M1) regressors, regions with significantly increased partial correlation were mainly located in contralesional parietal, prefrontal, and sensorimotor areas and regions with significantly decreased partial correlation were mainly observed in the ipsilesional supramarginal gyrus and superior temporal gyrus. Pearson's correlations revealed that the interhemispheric asymmetry change significantly correlated with the training effect as well as the integrity of M1-M1 anatomical connection. In summary, our study suggested that multiple functional brain regions not limited to motor areas were involved during the recovery process from multimodality perspective. The correlation analyses suggested the essential role of interhemispheric interaction in motor rehabilitation. Besides, the underlying structural substrate of the bilateral M1-M1 connection might relate to the interhemispheric change. This study might give some insights in understanding the neuroplasticity induced by the integrated BCI-guided robot hand training intervention and further facilitate the design of therapies for chronic stroke patients.

## 1. Introduction

Stroke is the leading cause of death worldwide, and the survivors undergo various disabilities related to motor, sensory, and cognitive functions. Specifically, robot-assisted therapy is a kind of task-specific and high-intensity exercise in an active, functional, and highly repetitive manner [1]. It has been proven to be efficient to induce neuroplasticity modulation and promising long-term motor recovery [2]. A brain computer interface (BCI) can facilitate stroke rehabilitation by integrating the exoskeleton robots to develop the BCI-guided robot-assisted therapy, which is believed to engage various brain functional regions [3] in the recovery process.

Electroencephalography (EEG), which can capture subtle neurological changes, has been widely used in studying neural functions. EEG signals result from the mixture of propagating electric potential fluctuations, mainly reflecting the postsynaptic activity of large populations of cortical pyramidal cells [4]. Additionally, functional magnetic resonance imaging (fMRI) has become one of the most commonly used neuroimaging tools to assess the cortical alterations associated with learning, diseases, or rehabilitation [5]. Resting-state fMRI that measures the temporal correlation of the blood oxygen level-dependent (BOLD) signal between different regions at resting state has emerged as a powerful tool to map the functional organization of the brain [6]. fMRI measurements have an excellent spatial resolution in millimeters but low temporal resolution limited to few seconds. While EEG holds millisecond-level temporal resolution, allowing the adequate sampling of the rapidly changing electrical dynamics of neuronal populations [4]. Since EEG and fMRI exhibit highly complementary characteristics, their integration has been widely exploited [7, 8]. Simultaneously recorded EEG and fMRI data make it possible to integrate these two neuroimaging modalities and have received substantial attention [9]. In our current study, we also adopted a concurrent EEG-fMRI technique to figure out related functional regions sensitive to the training effect. It should be noted that researchers have put numerous efforts to detecting these significant functional regions based on various neuroimaging techniques. For example, some studies have indicated the crucial role of supplementary motor area (SMA) in a motor system to execute various tasks including interlimb coordination [10] and many unimanual tasks involving movement sequencing as well as internal pacing [11]. Specifically, for stroke patients, the reduced partial correlation between SMA and M1 together with the interhemispheric correlation of both SMAs during visually paced hand movements has been found [12]. The reduced partial correlation between ipsilesional of SMA and M1 was also exhibited when stroke patients perform hand movements [13] and index finger-tapping task [14]. Meanwhile, it is noted that improved motor function of stroke patients might be highly correlated to a restitution of ipsilesional effective connectivity between SMA and M1 [15] and functional connectivity of the ipsilesional M1 with contralesional SMA [16]. Hence, in our study, we hypothesized SMA would also play an essential role in motor recovery with BCI-guided robot-hand training.

Quantification of EEG signal can be linked to the clinical features, such as the rehabilitation progress in chronic stroke. Nonetheless, due to the volume-conduction effect, the activities of scalp EEG signal are often assumed to come from multiple sources spatially dispersed in the brain cortex, which blurs the underlying neural mechanisms [17]. Therefore, EEG source imaging has emerged where the patient-specific anatomical properties could be taken into account using individual structural MRI images to disentangle useful neural information. However, few studies leveraged the indicators derived from EEG source space to investigate motor training effects for chronic stroke patients.

Although linear measurements have been widely recognized to reflect the brain characteristics, there is a growing tendency that different nonlinear measures have been proposed to depict the complexity of EEG signals and adopted to predict treatment response to repetitive transcranial magnetic stimulation in depression [18], evaluate the effect of stroke rehabilitation [19], and facilitate the classification system for hand recovery in stroke patients [20]. Fractal property that is quantified by the fractal dimension (FD) [21] is a nonlinear descriptor for brain signals, including EEG signals, which is closely associated with specialization and efficiency of brain functioning [22]. Investigation of such fractal nature as its correlation with the rehabilitation process for patients with neurological disorders, including stroke, is particularly important. The interhemispheric imbalance, especially the imbalance between homologous primary motor regions, always plays a crucial role in stroke motor rehabilitation [23]. Additionally, structural imaging, such as diffusion tensor imaging (DTI), has provided pivotal insights into the functional role of the underlying structural tracts in stroke-related changes [24]. Reductions in fractional anisotropy (FA), a DTI-derived measure of degree of anisotropy in white matter (WM), have been found in stroke individuals [25]. Specifically, the integrity of transcallosal motor fibers may play a role in monitoring the treatment response in chronic stroke [26].

The purpose of this study is to investigate the neural correlates of motor recovery after BCI-guided robot-assisted training in chronic stroke from a multimodality neuroimaging perspective. The EEG-informed fMRI analysis was utilized to locate the potential functional brain regions sensitive to the training effect. Furthermore, we hypothesized that the training effect should be related to the interhemispheric interaction change and such induced change was supposed to be based on the structural substrates connecting bilateral primary motor areas. Hence, the corresponding correlation analyses were performed to verify these hypotheses.

## 2. Materials and Methods

### 2.1. Subjects

Fourteen chronic stroke subjects (13 males, mean age = 54 ± 8 years, right-handedness) with unilateral hemispheric impairment were recruited from local community. The inclusion criteria were as follows: (1) first-ever stroke, (2) more than 6 months since the stroke onset prior to the experiment, (3) a single unilateral brain lesion, (4) Hong Kong Montreal Cognitive Assessment (HK-MoCA) [27, 28] score ≥ 22 to ensure sufficient cognitive function to understand instructions and perform tasks, (5) moderate to severe paretic hand dysfunctions (Fugl-Meyer Assessment score for upper extremity < 47), and (6) no additional rehabilitation therapies applied to the patient. The exclusion criteria were as follows: (1) aphasia, neglect, apraxia, history of alcohol, drug abuse, or epilepsy; (2) severe hand spasticity; (3) hand deformity and wound; and (4) severe cognitive deficits. Motor functions of the paretic upper limbs for all stroke subjects were assessed with Fugl-Meyer Assessment for upper extremity (FMA) which is a reliable and widely used measurement [29] before and after the intervention, respectively.  Table 1 summarizes the demographics and clinical properties of subjects.

### 2.2. Training Interventional Protocol

All subjects received a 20-session BCI-guided robot hand training therapy with an intensity of 3-5 sessions per week and completed the whole training process with 5-7 weeks. During each training session, the surface EEG signals of each subject were acquired using 16 electrodes (C1, C2, C3, C4, C5, C6, Cz, FC1, FC2, FC3, FC4, FCz, CP1, CP2, CP3, and CP4 according to international 10-20 system) at a sample rate of 256 Hz. The EEG signals were then amplified (g.LADYbird, g.USBamp, g.Tec Medical Engineering GmbH, Austria) and processed to generate the real-time topography of the brain electrical potential for surveillance. A paradigm with a fixed sequence showing instructions for motor imagery was played, during which the subjects were guided to imagine either grasping or releasing a cup following commands. EEG signal from C3 or C4 channel according to the subject's lesion side was extracted to calculate the *α* suppression [30]. The EEG data were transformed into the frequency domain using Fourier transform, and the mean power in the *α* band (8-13 Hz) was derived. Then, the *α* suppression was calculated as follows:

(1) $$\begin{array}{c} \alpha \text{ suppression}=\frac{P_{\text{rest}}-P_{\text{MI}}}{P_{\text{rest}}}, \end{array}$$

where *P*_MI_ and *P*_rest_ stand for the calculated *α* power during the motor imagery period and the resting-state, respectively. A trigger would be sent to an exoskeleton robot hand [31] (illustrated in Figure S1 B; a detailed description is provided in supplementary materials) to provide mechanical force and assist the paretic hand in grasping and opening if the *α* suppression exceeded 20% based on the previous study [32]. The success rate was defined as the percentage of correctly detected trials during motor imagery tasks at each session.

This study was approved by the Joint Chinese University of Hong Kong-New Territories East Cluster Clinical Research Ethics Committee. All subjects gave written consent before the intervention and underwent the experiments in the Chinese University of Hong Kong rehabilitation labs. This study was registered at https://clinicaltrials.gov (NCT02323061).

### 2.3. Data Acquisition

MRI scans were acquired for all the 14 subjects before and after the training sessions. A 3T Philips MR scanner (Achieva TX, Philips Medical System, Best, Netherlands) with an 8-channel head coil was used to acquire high-resolution T1-weighted anatomical images (TR/TE = 7.47/3.45 ms, flip angle = 8°, 308 slices, voxel size = 0.6 × 1.042 × 1.042mm^3^) using a T1-TFE sequence (ultrafast spoiled gradient echo pulse sequence), and BOLD fMRI images (TR/TE = 2000/30 ms, flip angle = 70°, 37 slices/volume, voxel size = 2.8 × 2.8 × 3.5mm^3^) using an EPI sequence (gradient-echo echo-planar-imaging sequence). Besides, diffusion-weighted images were acquired using a diffusion-weighted single-shot spin-echo echo-planar pulse (DWISE) sequence (TR/TE = 3788/88 ms, flip angle = 90° from 32 directions, 60 slices/volume, voxel size = 1.5 × 1.5 × 2mm^3^). When acquiring resting-state fMRI data, subjects were presented with a white cross in a black background and instructed to rest while focusing on the fixation cross. The resting-state fMRI acquisition lasted for 8 minutes.

The EEG data were acquired simultaneously with the fMRI using Neuroscan system (SynAmps2, Neuroscan Inc., Herndon, USA). A 64-channel MR-compatible EEG cap according to a standard 10-20 system was utilized, combined with 2 extra electrocardiogram (ECG) electrodes attached at the left lower and near-midline upper chest and 1 electrooculogram (EOG) electrode placed below the right eye. All recording impedances were kept below 5 k. The reference channel was located at the point between Cz and CPz; an AFz electrode was treated as the ground. Signals were filtered between 0.1 and 256 Hz using an analog filter and sampled at 1000 Hz for off-line processing. The whole scheme of experimental protocol is shown in Figure S1 A.

### 2.4. Data Analysis

**Figure 1:**
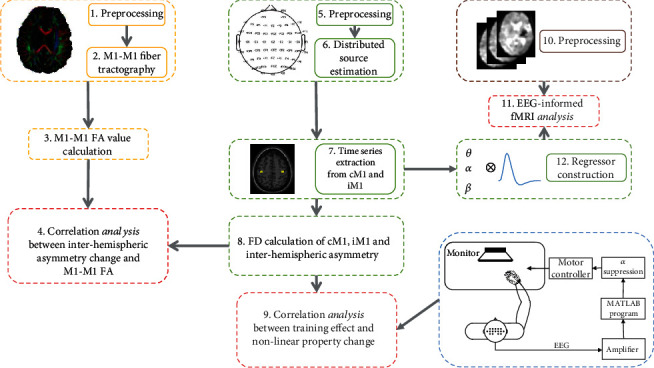
Illustration of analysis pipeline. The whole analysis included processing of fMRI, EEG, and DTI data; EEG-informed fMRI analysis; correlation analysis between training effect and nonlinear property change characterized by FD; and correlation analysis between interhemispheric asymmetry change and integrity of M1-M1 anatomical connection quantified by FA.

In our study, the analysis was mainly performed from multimodality perspective including fMRI, EEG, and DTI neuroimaging data, and the whole analysis pipeline is summarized in Figure 1. The left column included the preprocessing of DTI data, M1-M1 fiber tractography, and calculation of FA value of M1-M1 tract (please refer to section 2.7 in supplementary materials). The middle column included the analysis of EEG data including preprocessing, distributed source estimation, time series extraction from cM1 and iM1 seeds, and the calculation of indices characterizing nonlinear properties (please refer to sections 2.2, 2.3, and 2.6 in supplementary materials). The right column mainly included the preprocessing of fMRI data, iM1 EEG regressor construction, and integrated EEG-informed fMRI analysis (please refer to sections 2.1 and 2.4 in supplementary materials). The detailed description of each step is provided in the supplementary materials.

### 2.5. Statistical Analysis

Statistical analyses were performed using SPSS 25.0 (IBM SPSS Statistics, NY, US) with the significance level set at *p* < 0.05. A paired *t*-test between pretraining and posttraining was applied to examine if FMA score has changed after the intervention. For the conventional fMRI analysis, the paired *t*-test was performed for pre- and posttraining based on the individual partial correlation maps from 14 subjects. For EEG-informed fMRI analysis, two-way repeated-measure analysis of variance (ANOVA) was conducted with two fixed effects of time (pre- and posttraining) and frequency bands (theta, alpha, and beta) and with subject effect considered as a random effect for iM1 regressor. Paired *t*-tests as the post hoc analysis were further performed between pre- and posttraining based on the individual partial correlation maps for each frequency band. Multiple comparisons were corrected using Gaussian random field theory at the cluster level (voxel-wise significance: *p* < 0.005; cluster-wise significance: *p* < 0.05) [33]. For the survived clusters, false discovery rate (FDR) correction was further performed (*p* < 0.05) [34]. Pearson's correlation coefficients were calculated between FMA score changes and interhemispheric asymmetry change before and after the training. To investigate the potential underlying structural base influencing the interhemispheric property alternation, correlation analysis was also conducted between interhemispheric asymmetry change and FA of M1-M1 anatomical connection.

## 3. Result

We first assessed the effect of training on motor functions in the stroke participants. A paired *t*-test indicated a statistically significant improvement in FMA scores following training intervention, from 21 ± 6.7 to 25 ± 7 (*t*(13) = 3.313, *p* = 0.006). Besides, an increasing trend of success rate along with 20 training sessions was observed, with the mean of 73.01% for the first five sessions to 76.78% for the last five sessions.

**Figure 2:**
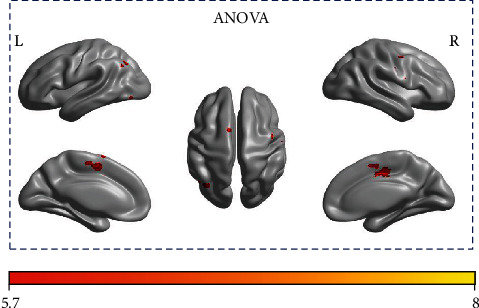
Surface rendering of brain functional regions which showed significant time × frequency interaction for iM1 regressor. The right side is the ipsilesional side.

The two-way ANOVA indicated that clusters with significant time × frequency interaction were found in a cluster at bilateral SMA, paracentral lobule and contralesional superior frontal gyrus (BA6 C&I), a cluster at ipsilesional precentral and postcentral gyrus (BA4 I and BA6 I), and a cluster at contralesional superior parietal lobe (SPL) and inferior parietal lobe (IPL) (BA7 C) (Illustrated in Figure 2 and summarized in Table 2).

**Figure 3:**
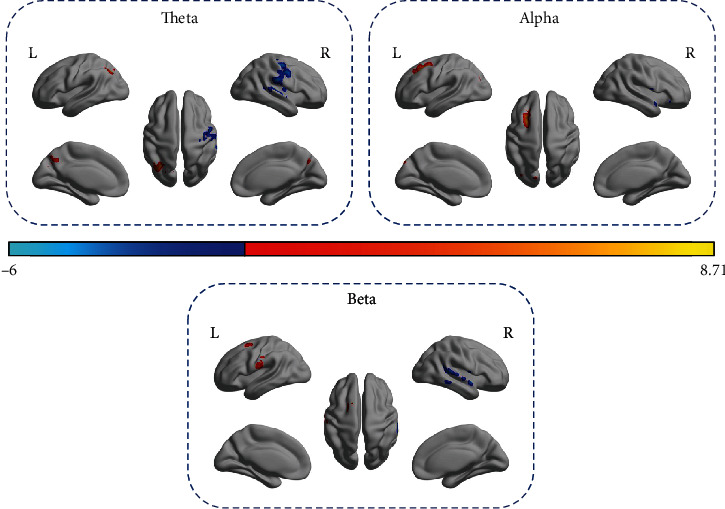
Surface rendering of brain functional regions which showed significant partial correlation change before and after training, given the regressor embedding spectral information (including theta, alpha, and beta frequency bands) derived from EEG source time course of iM1. The right side is the ipsilesional side.

Paired *t*-tests were performed between pre- and posttraining with different combinations of three representative EEG bands (theta, alpha, and beta). When theta band EEG signal was used as the regressor, significantly increased partial correlation was found in one cluster overlying the contralesional superior parietal gyrus, inferior parietal gyrus, and precuneus (BA7 C). Significantly decreased partial correlations were found in the ipsilesional precentral gyrus (BA4 I) and ipsilesional supramarginal gyrus (BA48 I). When alpha band EEG signal was used as the regressor, significantly increased partial correlations were found in a cluster involving the contralesional superior frontal gyrus and middle frontal gyrus (BA8 C and BA6 C) and the other clusters including the contralesional precuneus, cuneus, and superior occipital gyrus (BA7 C, BA19 C, and BA18 C). Significantly decreased partial correlation was found in the ipsilesional superior temporal gyrus (BA48 I). When beta band EEG signal was used as the regressor, significantly increased partial correlations were found in the contralesional postcentral gyrus (BA4 C) as well as a cluster covering contralesional SMA and superior frontal gyrus (BA6 C). Significantly decreased partial correlation was found in the ipsilesional superior temporal gyrus (BA48 I) (illustrated in Figure 3 and summarized in Table 3).

**Figure 4:**
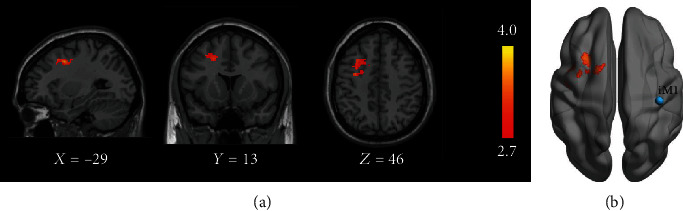
The conventional seed-based fMRI results illustrated from (a) sagittal view, coronal view, and axial view and (b) rendering surface. The right side is the ipsilesional side.

For conventional seed-based fMRI connectivity analysis, paired *t*-test showed that significant clusters were observed mainly in contralesional Brodmann area 6 when seed ROI was located at iM1 (illustrated in Figure 4).

**Figure 5:**
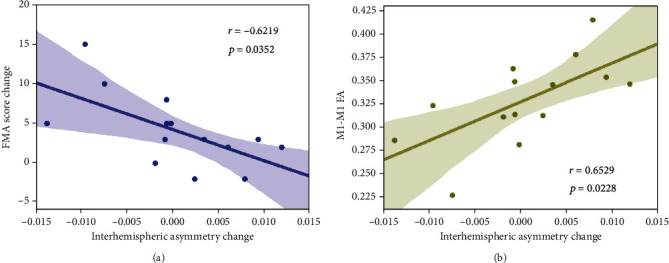
Significant correlations were observed (a) between FMA score change and interhemispheric asymmetry change as well as (b) between interhemispheric asymmetry change and FA of M1-M1 anatomical connection.

Then, we explored the relationship between training effect and nonlinear property changes quantified by FD. Pearson's correlation revealed that the FMA score change significantly correlated with interhemispheric asymmetry change (Figure 5(a), *r* = −0.6219, *p* = 0.0352; Bonferroni corrected) before and after training. We hypothesized that the alteration of interhemispheric property would be associated with corresponding structural characteristics. Indeed, a significant relevance between interhemispheric asymmetry change and FA of M1-M1 anatomical connection was observed (Figure 5(b), *r* = 0.6529, *p* = 0.0228; Bonferroni corrected).

## 4. Discussion

### 4.1. Motor Functional Recovery

The reorganization of the central nervous system plays an important role in the recovery of dysfunctions. It is an intrinsic property of the human brain to change its function and reorganize after a lesion forms [35], referred as neuroplasticity in stroke rehabilitation. Leveraging the mechanism of neuroplasticity, robot-assisted hand, which performs high-frequency movements accompanied by sensory feedback, has been shown to be an important factor in improving hand function [36]. The robotic hand could provide haptic as well as proprioceptive feedback on the intended movement. On the other hand, BCI is able to offer feedback to facilitate the appraisal of performance by enforcing the sensory aspect in the sensorimotor loop [37], thereby restoring the action-perception coupling. Some studies have observed significant improvement of FMA scores in BCI groups, but not in the control groups that receive random functional electrical stimulation (FES) [38] or receiving random robotic orthosis feedback [39]. Our study also showed the consistent significant FMA improvement after intervention involved BCI. Besides, the observed increasing trend of success rate also provided the evidence implying that the function of patients improved with more training sessions. In this context, a number of functional brain regions are expected to be involved in the process of recovery. It should be noted that not only perilesional but also distant brain regions would be affected even if the brain damage is focal [40, 41]. Hence, finding the regions responding to the effect induced by the training therapy is essential for a better understanding of the underlying mechanism of stroke recovery.

### 4.2. Related Functional Brain Regions

EEG oscillation has been used as an important index for evaluating brain activity, while different bands of EEG signals reflect various brain activities. EEG theta band has been associated with cognitive processing [42] and sensory stimulus identification and codification [43]. Alpha band oscillation is regarded as the dominant oscillatory activity of the human brain and has been associated with basic cognitive functions such as attention and memory [44]. Beta band oscillation is associated with a variety of processes, including top-down communication [45], sensory sampling [46], sensorimotor integration [47], and attention [48]. Simultaneous EEG-fMRI has been widely adopted to investigate how changes in electrophysiological oscillations may be linked to hemodynamic functional interactions within and between brain networks [49, 50].

**Figure 6:**
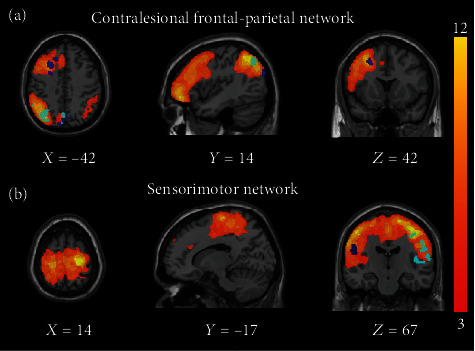
The overlap with contralesional frontal-parietal network and sensorimotor network. (a) The orange color-coded areas indicated the contralesional frontal-parietal network. The azure and violet color-coded areas indicated the regions which showed significant partial correlation change for theta and alpha band EEG signals from iM1. (b) The orange color-coded areas indicated the sensorimotor network. The azure and violet color-coded areas indicated the regions which showed significant partial correlation change for theta and beta band EEG signals from iM1. The contralesional frontal-parietal network and sensorimotor network were extracted using independent component analysis (ICA), and the detailed description of the extraction process is provided in supplementary materials. The right side is the ipsilesional side.

In our study, increased correlations with BOLD signal were observed in parietal regions for both theta and alpha band EEG signals from iM1 and in prefrontal regions for alpha band EEG signal from iM1. These regions overlapped with the well-known frontoparietal attention network, including portions of the lateral prefrontal cortex and posterior parietal cortex (illustrated in Figure 6(a)). The frontoparietal network is thought to be involved in a wide variety of tasks by initiating and modulating cognitive control abilities [51] and also regulating among default mode network, dorsal attention network, and central-executive networks [52, 53]. Previous simultaneous EEG-fMRI studies have reported that theta and alpha power from EEG correlated with BOLD signals from frontoparietal networks encompassing brain regions involved in an attention process [50, 54]. Meanwhile, it has been reported that brain regions in the frontal-parietal network are highly related to motor imagery BCI training [55] and correlate with the performance of MI-BCI [56]. Increased correlations with the BOLD signal were also observed in sensorimotor areas for beta band EEG signal from iM1 in our study (illustrated in Figure 6(b)). Mantini et al. indicated that the sensorimotor network is primarily associated with beta oscillations [50].

Interestingly, the observed increased correlations were all located in contralesional hemisphere, which suggested the crucial role of interactions between hemispheres, especially motor-related regions during a recovery process. A similar interhemispheric connectivity increase was also seen in fMRI studies. Longitudinal studies indicated that interhemispheric functional connectivity could predict motor improvements after stroke [15, 57]. Pichiorri et al. illustrated the more significantly increased interhemispheric connections between the ipsilesional motor area and contralesional frontal and parietal areas from the beta band of resting EEG data in the BCI-supported MI training group compared with the MI-only group, which were speculated as related to BCI training effects [58]. The similar increased interhemispheric partial correlation found in our study might also be related to BCI training effect. Furthermore, decreased correlations were observed in the ipsilesional supramarginal gyrus and superior temporal gyrus across all the frequency bands and in the ipsilesional precentral gyrus for theta band. It is also interesting to note that all corresponding regions were located in the ipsilesional hemisphere, which might be due to the close distance to the lesion, which implied the functional potential of intrahemispheric communication among ipsilesional regions, consistent with some previous studies [59]. Combining the regions found in the contralesional hemisphere, it seems to provide some evidence that impaired and intact hemispheres might play plausibly complementary roles in responding to training intervention, which deserves further investigation in the future.

For comparison, the conventional seed-based fMRI connectivity analysis was also conducted. It could be seen that most of the significant functional regions illustrated by the conventional seed-based fMRI connectivity analysis could also be detected by EEG-informed fMRI analysis whose regressors were reconstructed from EEG source signals in our study. Specifically, more functional regions derived from our proposed method were revealed to be influenced by the training intervention. We inferred that the reason for this phenomenon should be linked to high temporal resolution of EEG signal. This allowed more useful neural information to be disentangled from different frequency bands because there are abundant oscillatory activities in the human brain, which could provide a comprehensive understanding of the involvement of functional regions during the recovery process. It is worth noting that as we expected, the significant changes of the partial correlation with SMA were observed. The enhanced partial correlation between contralesional SMA and ipsilesional M1 was found in both conventional and EEG-informed fMRI analyses. Park and colleagues have shown that functional connectivity of ipsilesional M1 and contralesional SMA at onset was positively correlated with motor recovery at 6 months after stroke, which suggested the significance of preservation of such partial correlation [15]. Therefore, BCI-guided robot hand training might facilitate in restoring and enhancing the communication between contralesional SMA and ipsilesional M1, which might be beneficial for stroke motor recovery later.

### 4.3. Training Effect Correlated with Interhemispheric Interaction Change

It could be noted that the obvious pattern of interhemispheric interaction from a previous fMRI analysis result existed. We expected that such interhemispheric response should be closely related to training effect. There is a growing awareness about linking the potential motor function improvement after rehabilitation therapies with neural characteristics derived from electrophysiological signals to unfold the underlying mechanisms of the stroke recovery and treatment gains. The majority of these studies were focusing on linear indices, while nonlinear methods have drawn more attention recently. It has been indicated that the human brain is a nonlinear system [60], which cannot be comprehensively explained solely by linear analysis. The complex fluctuations of brain signals are not purely random but reveal a temporal organization over multiple time scales [61]. Hence, nonlinear methods have been proven to be efficient tools in understanding the complexities of the brain, and the measurement of EEG complexity could be linked to the efficiency of brain functional abilities [61]. On the other hand, different from fMRI, EEG has a higher temporal resolution and contains abundant nonlinear dynamic properties [62, 63]. Therefore, a variety of nonlinear methods have been applied to EEG analysis [64].

It was also widely believed that the presence of lesion following stroke would lead to an interhemispheric imbalance where iM1 no longer inhibited cM1 and the normal mutual communication between two hemispheres was severely broken, which has shown to positively correlate with motor impairment [65]. Therefore, the rebalance of the two hemispheres is essential for stroke rehabilitation and stroke recovery. It has been illustrated in some neuroimaging researches that increased change in resting-state functional connectivity of bilateral M1 coupled with better motor and functional improvements after robot-assisted bilateral arm therapy [23]. Pellegrino et al. found interhemispheric correlation changes correlated closely with the acquisition of more accurate hand control after robotic therapy [66]. Consistent with previous studies, our study also demonstrated the significant correlation between FMA score increment and decline in interhemispheric asymmetry, which indicated that more rebalance would bring about more motor improvement.

### 4.4. Structural Substrate of the Bilateral M1-M1 Connection

Due to the significance of interhemispheric rebalance in the recovery process, we hypothesized that such interhemispheric asymmetry change after training therapy should be built on some structural base in human brains, and the most intuitive one was the interhemispheric structural connectivity via transcallosal commissural projections. Hence, we further explored the association between corresponding asymmetry change and M1-M1 anatomical connection and found that the interhemispheric asymmetry change significantly correlated with the FA value of M1-M1 connection fibers, which indicated that more interhemispheric rebalance could be achieved for patients with lower M1-M1 anatomical connection. In a recent study, it was observed that, among stroke patients with good motor outcomes, those with more severe impairment in M1-M1 anatomical connection had a higher M1-M1 resting-state functional connectivity [67] which implied the importance of restoring interhemispheric interaction for patients with lower M1-M1 connection level to achieve ultimate recovery goal. Together, these findings suggested that our training intervention protocols or similar therapies should be considered, especially for patients with poor M1-M1 anatomical connection.

### 4.5. Limitations and Future Work

Several limitations need to be noted in the current study. First of all, the sample size was not large, which might limit the generalization power to some extent. Second, most of our subjects were male which might restrict our finding extended to female stroke population although we assumed that the gender factor was less likely to affect the result significantly. Another potential concern was about the influence of the stroke lesion on the reconstructed source data. It should be noted that, in the current cohort of stroke subjects, most lesions were located in the subcortical regions. Because of this, we limited the source space to the cortex when performing the EEG distributed source estimation which was also widely adopted in practice [68]. Meanwhile, we mainly extracted source signals from iM1 and cM1 seeds which were also far away from the lesion regions. Besides, the 64-channel EEG set-up could already achieve an accurate description of the spatial distribution of the stroke-related EEG, which guaranteed the quality of the source estimation to some extent [69]. Hence, in our study, the influence of the stroke lesion on the quality of the reconstructed source data was supposed to be negligible. However, more advanced algorithms that take the brain lesions into account could be developed for more accurate source estimation in future studies. Besides, due to the lack of the control condition, it is quite difficult to check whether and to what extent the observations were clearly linked to our experimental intervention. Therefore, a control group with pure robot hand training without BCI could be included to clarify this vagueness in the future.

Recently, studies have proposed that surface-based methods might improve the quality of cortical area localization compared to the volume-based methods in fMRI analysis [70]. However, it should be noted that studies on EEG-informed fMRI with a surface-based method are quite scarce, compared with a volume-based method. We also tried a preliminary attempt on EEG-informed fMRI analysis with surface-based method. The detailed analysis process and the observed results were described in supplementary materials. Furthermore, a more standard pipeline of EEG-informed fMRI analysis with surface-based method should be developed to fill this gap in the future.

## 5. Conclusion

This study presented a paradigm to investigate the neural correlates of motor recovery after training therapy based on multimodality neuroimaging techniques, which could provide more complementary information for each other such as oscillatory information derived from EEG signal. Some significant brain regions linked to important functional networks were observed to be sensitive to our integrated BCI-guided robot-hand training intervention, although cautions should be taken when interpreting these observations due to the absence of a control group. The training effect was found to be highly related to interhemispheric asymmetry alternation. The underlying structural substrate might be associated with M1-M1 anatomical connection. Finally, our study provided valuable clinical information for both stroke prognosis and understanding of regional communication in the brain given training therapy.

## Figures and Tables

**Table 1:** Demographics and clinical properties of the participants.

No.	Age range	Gender	Stroke onset (years)	Lesion side	Lesion location	Stroke type	FMA (Max score: 66)	
							Pre	Post
S1	45-49	M	1	R	MFG, SFG, precentral, supramarginal, SMA	Ischemic	19	34
S2	65-69	M	8	L	Insula, putamen, IFG, temporal pole	Hemorrhage	22	27
S3	65-69	M	1	R	Insula, ITG, IOG, putamen	Hemorrhage	13	16
S4	60-64	M	3	R	Insula, putamen, IFG Rolandic operculum	Ischemic	16	14
S5	45-49	M	0.7	R	ITG, MTG, STG, MOG, angular, supramarginal	Hemorrhage	17	25
S6	60-64	M	11	L	PLIC, putamen, insula, postcentral, SFG	Ischemic	22	24
S7	55-59	M	6	R	Insula, IFG Rolandic operculum	Ischemic	13	23
S8	40-44	M	5	R	Insula, Rolandic operculum, IFG, STG, putamen, temporal pole	Hemorrhage	15	17
S9	50-54	F	3	L	Insula, Rolandic operculum, putamen	Hemorrhage	34	34
S10	40-44	M	3	R	Insula, MTG, STG, temporal pole, putamen, Rolandic operculum	Hemorrhage	17	20
S11	55-59	M	5	L	Insula, IFG, putamen	Hemorrhage	28	33
S12	50-54	M	1	L	Putamen, caudate nucleus	Ischemic	24	22
S13	55-59	M	7	R	Putamen, temporal pole, IFG, insula, Rolandic operculum	Ischemic	20	25
S14	45-49	M	1	R	Insula, putamen	Hemorrhage	34	37

F: female; FMA: Fugl-Meyer Assessment for upper limb; IFG: inferior frontal gyrus; IOG: inferior occipital gyrus; ITG: inferior temporal gyrus; L: left hemisphere lesion; M: male; MFG: middle frontal gyrus; MOG: middle occipital gyrus; MTG: middle temporal gyrus; PLIC: posterior limb of the internal capsule; SFG: superior frontal gyrus; SMA: supplementary motor area; STG: superior temporal gyrus; R: right hemisphere lesion.

**Table 2:** Brain regions showing significant time × frequency interaction.

C/I	Anatomical region	Peak MNI coordinate (*x*, *y*, *z*)		
iM1 regressor				
C&I	Supplementary motor area	-5	1	48
C&I	Paracentral lobule			
C	Superior frontal gyrus			
I	Precentral gyrus	60	8	26
I	Postcentral gyrus			
C	Superior parietal lobe	60	8	26
C	Inferior parietal lobe			

C/I: contralesional or ipsilesional.

**Table 3:** Brain regions showing significant pre-post partial correlation change.

Frequency band	+/-	C/I	Anatomical region	Peak MNI coordinate (*x*, *y*, *z*)		
iM1 regressor						
Theta	+	C	Superior parietal gyrus	-32	-62	40
		C	Inferior parietal gyrus			
		C	Precuneus			
	-	I	Precentral gyrus	52	-16	26
		I	Supramarginal gyrus	46	-11	49
Alpha	+	C	Superior frontal gyrus	-24	18	42
		C	Middle frontal gyrus			
		C	Precuneus	-8	-86	38
		C	Cuneus			
		C	Superior occipital gyrus			
	-	I	Temporal pole	58	10	0
Beta	+	C	Postcentral gyrus	-54	-14	24
		C	Supplementary motor area	-12	8	56
		C	Superior frontal gyrus			
	-	I	Superior temporal gyrus	67	-20	12

**+/-**: increased or decreased; C/I: contralesional or ipsilesional.

## Data Availability

The data generated for this study are available from the corresponding author on reasonable request.

## Supplementary Materials

Supplementary Materials Supplementary materials consist of section 1—Scheme of Experimental Protocol and Photograph of the Robot Hand, section 2—Data Analysis, section 3—Extraction of Contralesional Frontal-Parietal Network and Sensorimotor Network, section 4—Interhemispheric Asymmetry before and after Training, and section 5—EEG-Informed fMRI Analysis using Surface-Based Method.
