# Supplementary material for: Neural Correlates of Motor Recovery after Robot-Assisted Training in Chronic Stroke: A Multimodal Neuroimaging Study
Source: Neural Plast. 2021 Jun 9;2021:8866613. doi: 10.1155/2021/8866613 (PMC8208881; doi:10.1155/2021/8866613)
Supplement: Supplementary Materials — consist of section 1—Scheme of Experimental Protocol and Photograph of the Robot Hand, section 2—Data Analysis, section 3—Extraction of Contralesional Frontal-Parietal Network and Sensorimotor Network, section 4—Interhemispheric Asymmetry before and after Training, and section 5—EEG-Informed fMRI Analysis using Surface-Based Method. [file 8866613.f1.pdf]

Supplementary materials: Neural correlates of motor recovery after robot-assisted training in chronic stroke: A multimodal neuroimaging study

Cheng Chen[1] ‡, Kai Yuan[1] ‡, Xin Wang[1], Ahsan Khan[1], Winnie Chiu-wing Chu[2], Raymond Kai-yu Tong[1]

[1]Department of Biomedical Engineering, The Chinese University of Hong Kong, Shatin, Hong Kong, [2] Department of Imaging and Interventional Radiology, The Chinese University of Hong Kong, Shatin, Hong Kong.

‡ ***Equal contributor.***

1. Scheme of experimental protocol and photograph of the robot hand

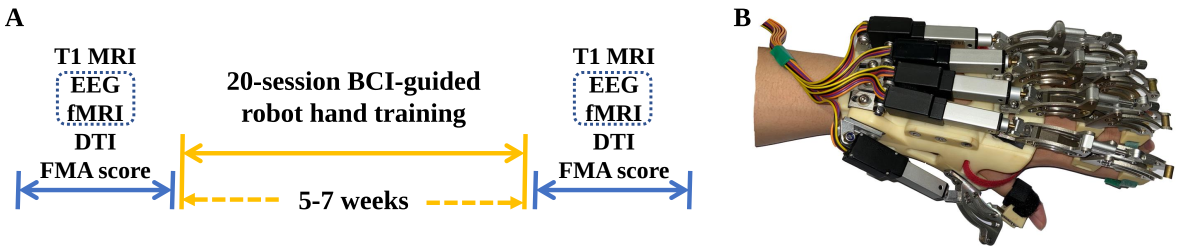

Figure S1: (A) Scheme of the experimental protocol showing the time of various data collection as well as the training therapy. The dashed block means the EEG and fMRI data were collected simultaneously. (B) Photograph of the robot hand used in the study.

The robot hand could provide 55 and 65 degrees range of motion (ROM) for metacarpophalangeal (MCP) and interphalangeal (PIP) finger joints respectively from fully extended position to fully flexed position. It took around 2 seconds to finish fully open or close movement under empty load. The subjects were instructed to keep their shoulders naturally flexed and abducted, elbow flexed at 90 degrees, arm pronated but wrist positioned naturally without any flexion or extension.

2. Data Analysis

2.1. fMRI Preprocessing

The resting-state fMRI data were preprocessed using the Data Processing Assistant for Resting-State fMRI (DPARSF) toolbox [1] based on Statistical Parametric Mapping (SPM12) (http://www.fil.ion.ucl.ac.uk/spm). The first 10 volumes were discarded to assure the remaining volumes of fMRI data were at magnetization steady state. The remaining volumes were corrected with slice timing and realigned for head motion correction. Nuisance variables were then regressed out, including white matter, cerebrospinal fluid (CSF), global mean signal, and Friston 24 head motion parameters [2]. To further control for head motion, the scrubbing process was performed for the volumes with frame-wise displacement value exceeding 0.3 [3]. If over 25% of all the volumes exceed the frame-wise displacement threshold, the data for this subject would be discarded, and no subject was discarded in resting-state fMRI analysis. Then the functional dataset was aligned to the anatomical dataset. Detrending and temporal band-pass filtering (0.01-0.1 Hz) [4, 5] were performed to remove higher frequency physiological noise and lower frequency scanner drift. Subsequently, the functional images were spatially normalized to the Montreal Neurological Institute (MNI) template, resliced to 2×2×2 mm^3 voxels, and smoothed with a Gaussian kernel with a full-width at half-maximum (FWHM) of 6 mm. In the statistical analysis, subjects

with left-hemispheric lesions were flipped along the midsagittal plane using MRIcron (www.mccauslandcenter.sc.edu/mricro/mricron). This procedure was also adopted in previous studies [6, 7], so that the lesions of all subjects were in the right hemisphere. (Corresponding to step 10 in Figure 1.)

2.2. EEG Preprocessing

EEG data were mainly preprocessed with EEGLAB [8], Fieldtrip toolbox [9], Matlab signal processing toolbox and custom-made codes (Mathworks Natick, MA, USA). The EEG signal had a relatively low signal-to-noise ratio (SNR) when simultaneously collected with fMRI due to the switching of magnetic field gradients. Therefore, a principal component analysis (PCA)-based optimal basis set (OBS) algorithm [10] was utilized to remove the MRI gradient artifact. The triggers marking the onset of each fMRI volume were also provided to better select and extract the artifactual features. In our study, the missing triggers for two subjects were repaired manually by an expert radiographer. The resulting EEG signal was inspected again to ensure no massive residual gradient artifact was observed. Ballistocardiographic (BCG) artifact related to heart pulse was another source distorting the EEG. To remove the artifact, the QRS complexes of each heart pulse were initially automatically determined with an R-peak detection algorithm. Then, a strategy developed by Liu and colleagues, which combined independent component analysis (ICA), OBS and an information-theoretic rejection criterion, was adopted to eliminate the BCG artifact channel-wisely [11]. After gradient and BCG artifact removal, the EEG signals were resampled to 250 Hz and further band-pass filtered with a 2-40 Hz Butterworth non-causal filter. Bad channels with extensive artifacts were removed and reconstructed through spherical spline interpolation with neighbor electrodes. All data were then common average referenced. Subsequently, the EEG data were segmented into non-overlapping two-second epochs according to the triggers from MRI scanner. The first and last four epochs were dropped, considering the signal instability. The remaining epochs were inspected visually accompanied by statistical metrics (e.g., z-score, variance, min and max, etc.), and bad epochs were rejected. Finally, an adaptive mixture independent component analysis (AMICA) algorithm [12] was utilized to wipe off the components potentially related to residual artifacts induced by MRI scanning, eye movements, and muscle contraction. The time courses of remaining components were projected back to all channels. To run appropriately the group-level analysis, EEG data for patients with left-hemispheric lesions were left-right flipped along the middle line before signal processing procedures. (Corresponding to step 5 in Figure 1.)

2.3. EEG distributed source estimation

The preprocessed EEG data were reconstructed into the distributed source space using Brainstorm software [13]. First, EEG electrode positions were manually co-registered to the individual T1 images based on the standard 10-20 EEG electrode locations and

polygon models of the cortical surface were constructed. The three realistic layers (scalp, inner and outer skull) were extracted from individual MRI data to generate the boundary element model (BEM) surface. The gain matrix, called the lead field, representing the linear relationship projecting the source time courses on the cortical surface to the measurements on EEG channels, was computed using OpenMEEG [14]. The Tikhonov-regularized minimum-norm algorithm was adopted to perform EEG source localization [15], with Tikhonov parameter which regularized the smoothness of the images set to 10% of maximum singular value of the lead field. Herein, one constraint was imposed that only a single current dipole was assumed at each vertex point and oriented normally to the cortical surface based on the anatomical observation. The noise covariance matrix was calculated over a long period of recordings, as recommended by Tadel and colleagues [13]. (Corresponding to step 6 in Figure 1.)

To investigate the neural activities from primary motor areas, two spherical seeds with a radius of 5 mm at ipsilesional M1 (iM1) and contralesional M1 (cM1) were determined. The iM1 and cM1 seeds were set at (38,-22,56) and (-38,-22,56) in the MNI space, respectively. The standard seeds in MNI space were wrapped to subject individual cortex space and the time series of vertices within the seed were averaged. The extracted time series from iM1 and cM1 seeds were mainly explored in further analysis. (Corresponding to step 7 in Figure 1.)

2.4. EEG-informed fMRI analysis

The extracted EEG source time courses in iM1 seed for each subject were further used in EEG-informed fMRI analysis [16, 17]. Time-frequency power spectrum was estimated for each extracted EEG source time course using short-time Fourier transform and the length of the sliding window was 2 seconds without overlap between adjacent windows. The EEG bands were defined as theta (4-7 Hz), alpha (8–12 Hz), and beta (13–30 Hz). Band power signals were calculated by averaging the power spectrum within each EEG band. The band power signals were further convolved with the hemodynamic response function (HRF) in SPM12, to obtain the EEG regressors of interest in partial correlation analysis [18, 19]. (Corresponding to step 12 in Figure 1.)

For each EEG band and each subject, general linear models (GLM) were fitted for every voxel in the brain with the corresponding EEG regressor and six motion parameters in fMRI analysis as covariables to control the effects of head motion. The GLM model was formed as follows [20]:

$$\boldsymbol{Y} = \boldsymbol{X}\boldsymbol{\beta} + \epsilon \tag{1}$$

where $\boldsymbol{Y}$ represented the measured fMRI signal of a single voxel. $\boldsymbol{X}$ was the design matrix. In this study, the first column of $\boldsymbol{X}$ was EEG regressor and the remaining ones were composed of six motion parameters. $\boldsymbol{\beta}$ was the weighting vector which was to be estimated and the entry corresponding to the EEG regressor would be treated as the partial correlation for this voxel. $\boldsymbol{\epsilon}$ was the random error term. The map of partial

correlation would be produced after traversing all voxels. (Corresponding to step 11 in Figure 1.)

2.5. Conventional seed-based fMRI connectivity analysis

Except for EEG-informed fMRI analysis, conventional seed-based fMRI functional whole-brain connectivity analysis was also performed. The same seeds of iM1 in MNI space were used, and the average time course of the BOLD signal within the seeds was calculated as the regressors. Following that, functional connectivity maps were generated.

2.6. Fractal Analysis

The fractal analysis on the iM1 and cM1 source time series was characterized by fractal dimension (FD) [21], a measure which has been shown to be able to capture the non-linear information on scale-free properties and to extend the understanding of brain mechanisms at system levels [22, 23]. Usually, brain damage could result in less complexity of the underlying neuronal activity, which is indicated in the decrease of FD value. Plenty of algorithms are available to calculate the FD and box-counting method [24] is a classical one among them. In our study, we adopted a strategy similar to Raghavendra and Dutt [25] to increase the box size from the finest time resolution to the maximum coarse time resolution with the increasing factor equal to two. The FD values were calculated in the 10-second sliding time windows with 50% overlap and averaged over all windows.

The balance of homologous motor regions is of importance in rehabilitation for stroke patients with motor impairment. In order to investigate the complexity unbalance, $FD_{asymmetry}$ was computed to characterize the interhemispheric asymmetry of motor areas as follows:

$$FD_{asymmetry} = \frac{FD_{cM1} - FD_{iM1}}{FD_{cM1} + FD_{iM1}} \tag{2}$$

where FD_{cM1} and FD_{iM1} are FD values for cM1 and iM1 respectively. In this study, we utilized the pre-post change of FD asymmetry to characterize the process of interhemispheric rebalance after the training intervention. (Corresponding to step 8 in Figure 1.)

2.7. DTI analysis

Diffusion-weighted images before the training were used for further analysis and first preprocessed using FMRIB Software Library (FSL), including correction for eddy-current effect and brain extraction. Diffusion tensors were then estimated from the preprocessed diffusion images. Fractional anisotropy (FA) [26], a measure quantifying the degree of anisotropic diffusion, was calculated from the diffusion tensors of each voxel. All the diffusion images were registered to MNI space. To assess the transcallosal

connections between two hemispheres, especially the reserved connection between primary motor areas, a recently published standard template TCATT (Transcallosal Tract Template) [27] was used to map the structural integrity. Average FA values within the M1-M1 tract were computed as the structural integrity between the bilateral motor cortex. (Corresponding to step 1, 2 and 3 in Figure 1.)

3. Extraction of contralesional frontal-parietal network and sensorimotor network

The contralesional frontal-parietal network and sensorimotor network were extracted using independent component analysis (ICA). All fMRI scans from all sessions were combined and run in a group ICA using the temporal concatenation method implemented in MELODIC [28], constrained to identify 30 components. An initial analysis of the population using model order estimation was used to calculate the appropriate number of components. The contralesional frontal-parietal network and sensorimotor network were chosen based on previous studies [29].

4. Interhemispheric asymmetry before and after training

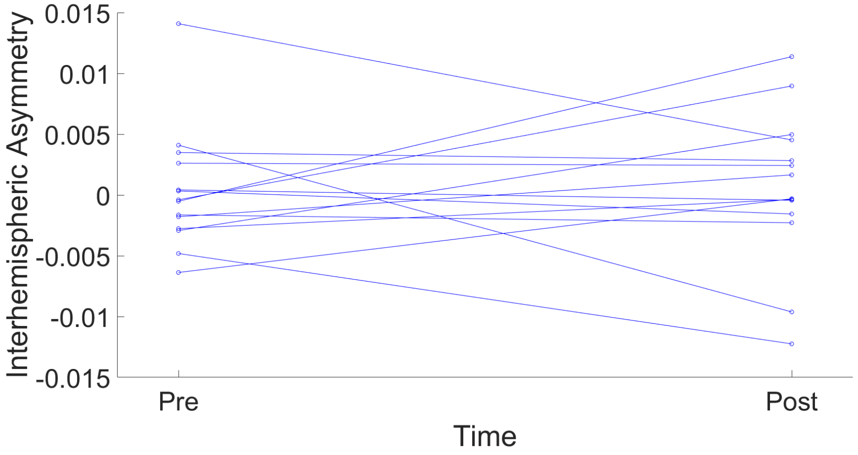

Figure S2: The interhemispheric asymmetry values before and after training for each individual subject.

5. EEG-informed fMRI analysis using surface-based method

MRI data were preprocessed using the fMRIPrep software version 1.5.0 [30]. T1-weighted images were corrected for intensity non-uniformity [31] and skull-stripped using ANTS 2.2.0 [32]. High resolution cortical surfaces were reconstructed with FreeSurfer 6.0.1 [33] based on T1-weighted images, and then registered to the fsaverage5 template

[34]. Functional data was slice-time corrected using 3dTshift [35], motion corrected using MCFLIRT [36], distortion corrected, and then resampled to the fsaverage5 template based on boundary-based registration [37]. Nuisance variables were then regressed out, including white matter, cerebrospinal fluid (CSF), global mean signal, and Friston 24 head motion parameters [2]. The surface data were further smoothed within 6 vertices.

Similar EEG-informed fMRI analysis as the volume-based analysis was performed on the preprocessed surface data. The two-way ANOVA with time (pre and post) and frequency bands as factors were performed. Vertex-level threshold was set at $p < 0.005$. No significant interaction result was found for iM1 seed. To compare with the volume-based results, paired t-tests were performed between pre and post training for 3 frequency bands (theta, alpha, and beta) and two hemispheres. Vertex-level threshold was set at $p < 0.005$. Significant clusters were only observed for alpha-band and theta-band for the iM1 seed. Consistent with the volume-based results, significant increased partial correlation was observed in the contralesional superior frontal area for alpha-band, and significant decreased partial correlation was observed in the ipsilesional supramarginal gyrus for theta-band. Besides, significant increased partial correlation was also observed in the contralesional superior frontal area for the theta-band. (illurstrated in Figure S3)

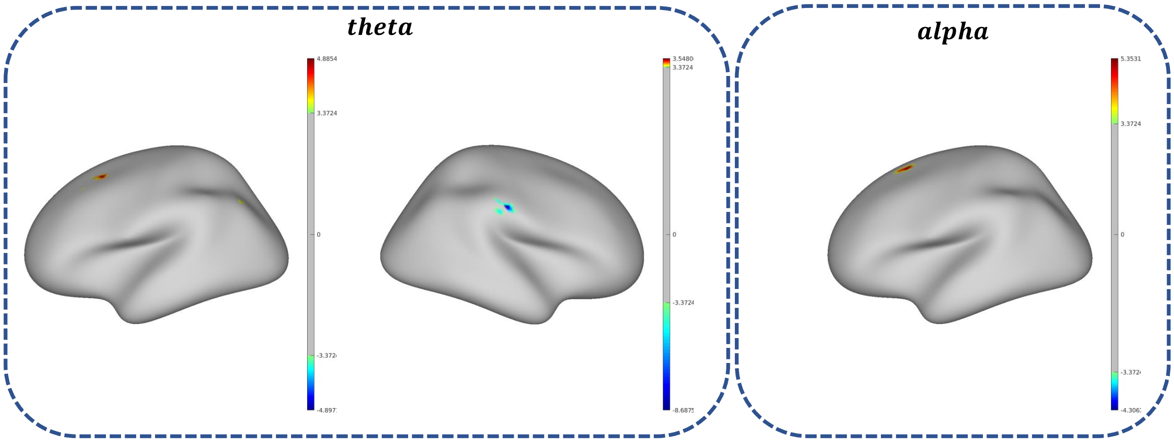

Figure S3: The brain regions which showed significant partial correlation change before and after training using surface-based method, given the regressor embedding spectral information (including theta, alpha frequency bands) derived from EEG source time course of iM1.

Reference

[1] C.-G. Yan, X.-D. Wang, X.-N. Zuo, and Y.-F. Zang, "Dpabi: Data processing & analysis for (resting-state) brain imaging," *Neuroinformatics*, vol. 14, pp. 339–351, Jul 2016.

[2] K. J. Friston, S. Williams, R. Howard, R. S. J. Frackowiak, and R. Turner, "Movement-related effects in fmri time-series," *Magnetic Resonance in Medicine*, vol. 35, no. 3, pp. 346–355, 1996.

[3] J. D. Power, K. A. Barnes, A. Z. Snyder, B. L. Schlaggar, and S. E. Petersen, "Spurious but systematic correlations in functional connectivity mri networks arise from subject motion," *NeuroImage*, vol. 59, no. 3, pp. 2142 – 2154, 2012.

[4] D. P. Auer, "Spontaneous low-frequency blood oxygenation level-dependent fluctuations and functional connectivity analysis of the 'resting' brain," *Magnetic Resonance Imaging*, vol. 26, no. 7, pp. 1055 – 1064, 2008. Proceedings of the International School on Magnetic Resonance and Brain Function.

[5] X.-N. Zuo, A. D. Martino, C. Kelly, Z. E. Shehzad, D. G. Gee, D. F. Klein, F. X. Castellanos, B. B. Biswal, and M. P. Milham, "The oscillating brain: Complex and reliable," *NeuroImage*, vol. 49, no. 2, pp. 1432 – 1445, 2010.

[6] N. Ward, M. Brown, A. Thompson, and R. Frackowiak, "Neural correlates of outcome after stroke: a cross-sectional fmri study," *Brain : a journal of neurology*, vol. 126, pp. 1430–48, 07 2003.

[7] X. Hong, Z. Lu, I. Teh, F. Nasrallah, W.-P. Teo, K. Ang, K. S. Phua, C. Guan, E. Chew, and K.-H. Chuang, "Brain plasticity following mi-bci training combined with tdcs in a randomized trial in chronic subcortical stroke subjects: A preliminary study," *Scientific Reports*, vol. 7, p. 9222, 08 2017.

[8] A. Delorme and S. Makeig, "Eeglab: an open source toolbox for analysis of single-trial eeg dynamics including independent component analysis," *Journal of Neuroscience Methods*, vol. 134, no. 1, pp. 9 – 21, 2004.

[9] R. Oostenveld, P. Fries, E. Maris, and J.-M. Schoffelen, "Fieldtrip: Open source software for advanced analysis of meg, eeg, and invasive electrophysiological data," *Computational intelligence and neuroscience*, vol. 2011, p. 156869, 01 2011.

[10] R. Niazy, C. Beckmann, G. Iannetti, J. Brady, and S. Smith, "Removal of fmri environment artifacts from eeg data using optimal basis sets," *NeuroImage*, vol. 28, no. 3, pp. 720 – 737, 2005.

[11] Z. Liu, J. A. de Zwart, P. van Gelderen, L.-W. Kuo, and J. H. Duyn, "Statistical feature extraction for artifact removal from concurrent fmri-eeg recordings," *NeuroImage*, vol. 59, no. 3, pp. 2073 – 2087, 2012.

[12] J. A. Palmer, K. Kreutz-Delgado, and S. Makeig, "Super-gaussian mixture source model for ica," in *Independent Component Analysis and Blind Signal Separation* (J. Rosca, D. Erdogmus, J. C. Príncipe, and S. Haykin, eds.), (Berlin, Heidelberg), pp. 854–861, Springer Berlin Heidelberg, 2006.

[13] F. Tadel, S. Baillet, J. Mosher, D. Pantazis, and R. Leahy, "Brainstorm: A user-friendly application for meg/eeg analysis," *Computational intelligence and neuroscience*, vol. 2011, p. 879716, 04 2011.

[14] A. Gramfort, T. Papadopoulo, E. Olivi, and M. Clerc, "Openmeeg: Opensource software for quasistatic bioelectromagnetics," *Biomedical engineering online*, vol. 9, p. 45, 09 2010.

[15] S. Baillet, J. Mosher, and R. Leahy, "Electromagnetic brain mapping," *Signal Processing Magazine, IEEE*, vol. 18, pp. 14 – 30, 12 2001.

[16] E. Formaggio, S. F. Storti, A. Bertoldo, P. Manganotti, A. Fiaschi, and G. M. Toffolo, "Integrating eeg and fmri in epilepsy," *NeuroImage*, vol. 54, no. 4, pp. 2719 – 2731, 2011.

[17] J. de Munck, S. Gonçalves, R. Mammoliti, R. Heethaar, and F. L. da Silva, "Interactions between different eeg frequency bands and their effect on alpha–fmri correlations," *NeuroImage*, vol. 47, no. 1, pp. 69 – 76, 2009.

[18] R. Abreu, A. Leal, and P. Figueiredo, "Eeg-informed fmri: A review of data analysis methods," *Frontiers in Human Neuroscience*, vol. 12, p. 29, 2018.

[19] T. Murta, M. Leite, D. W. Carmichael, P. Figueiredo, and L. Lemieux, "Electrophysiological correlates of the bold signal for eeg-informed fmri," *Human Brain Mapping*, vol. 36, no. 1, pp. 391–414, 2015.

[20] M. Monti, "Statistical analysis of fmri time-series: A critical review of the glm approach," *Frontiers in human neuroscience*, vol. 5, p. 28, 03 2011.

[21] A. Di Ieva, F. Grizzi, H. Jelinek, A. Pellionisz, and G. Losa, "Fractals in the neurosciences, part i: General principles and basic neurosciences," *Neuroscientist*, vol. 2013 December, 12 2013.

[22] A. D. Ieva, F. Grizzi, H. Jelinek, A. J. Pellionisz, and G. A. Losa, "Fractals in the neurosciences, part i: General principles and basic neurosciences," *The Neuroscientist*, vol. 20, no. 4, pp. 403–417, 2014. PMID: 24362815.

[23] M. Le Van Quyen, V. Navarro, J. Martinerie, M. Baulac, and F. Varela, "Toward a neurodynamical understanding of ictogenesis," *Epilepsia*, vol. 44 Suppl 12, pp. 30–43, 02 2003.

[24] J. Feder, "Fractals," *Plenum Press*, 1988.

[25] B. Raghavendra and D. Dutt, "Computing fractal dimension of signals using multiresolution box-counting method," *World Academy of Science, Engineering and Technology*, vol. 37, pp. 1266–1281, 01 2010.

[26] P. Basser and D. Jones, "Diffusion-tensor mri: Theory, experimental design and data analysis - a technical review," *NMR in biomedicine*, vol. 15, pp. 456–67, 11 2002.

[27] D. B. Archer, S. A. Coombes, N. R. McFarland, S. T. DeKosky, and D. E. Vaillancourt, "Development of a transcallosal tractography template and its application to dementia," *NeuroImage*, vol. 200, pp. 302 – 312, 2019.

[28] U. Amadi, A. Ilie, H. Johansen-Berg, and C. Stagg, "Polarity-specific effects of motor transcranial direct current stimulation on mari resting state networks," *NeuroImage*, vol. 88, 11 2013.

[29] C. Beckmann, M. DeLuca, J. Devlin, and S. Smith, "Investigations into resting-state connectivity using independent component analysis," *Philosophical transactions of the Royal Society of London. Series B, Biological sciences*, vol. 360, pp. 1001–13, 06 2005.

[30] O. Esteban, C. Markiewicz, R. Blair, C. Moodie, A. Isik, A. Erramuzpe, J. Kent, M. Goncalves, E. DuPre, M. Snyder, H. Oya, S. Ghosh, J. Wright, J. Durnez, R. Poldrack, and K. Gorgolewski, "fmriprep: a robust preprocessing pipeline for functional mri," *Nature Methods*, vol. 16, 01 2019.

[31] N. Tustison, B. Avants, P. Cook, Y. Zheng, A. Egan, P. Yushkevich, and J. Gee, "N4itk: improved n3 bias correction," *Medical Imaging, IEEE Transactions on*, vol. 29, pp. 1310 – 1320, 07 2010.

[32] B. Avants, C. Epstein, M. Grossman, and J. Gee, "Symmetric diffeomorphic image registration with cross-correlation: Evaluating automated labeling of elderly and neurodegenerative brain," *Medical image analysis*, vol. 12, pp. 26–41, 03 2008.

[33] B. Fischl, "Freesurfer," *NeuroImage*, vol. 62, no. 2, pp. 774–781, 2012. 20 YEARS OF fMRI.

[34] B. Fischl, M. Sereno, and A. Dale, "Cortical surface-based analysis: Ii: Inflation, flattening, and a surface-based coordinate system," *NeuroImage*, vol. 9, pp. 195–207, 03 1999.

[35] R. Cox, "Afni: Software for analysis and visualization of functional magnetic resonance neuroimages," *Computers and biomedical research, an international journal*, vol. 29, pp. 162–73, 07 1996.

[36] M. Jenkinson, P. Bannister, M. Brady, and S. Smith, "Improved optimization for the robust and accurate linear registration and motion correction of brain images," *NeuroImage*, vol. 17, pp. 825–41, 11 2002.

[37] D. Greve and B. Fischl, "Accurate and robust brain image alignment using boundary-based registration," *NeuroImage*, vol. 48, pp. 63–72, 07 2009.
